# Delayed right ventricular lead perforation by a pacemaker lead 2‐year post‐implantation

**DOI:** 10.1002/ccr3.5760

**Published:** 2022-04-18

**Authors:** Akihiro Yamamoto, Shoichi Takahashi

**Affiliations:** ^1^ Department of Cardiovascular Surgery Hoshi general hospital Fukushima Japan

**Keywords:** cardiac tamponade, delayed perforation, non‐infections, pacemaker lead

## Abstract

Cardiac perforation by the lead of cardiac implantable electronic devices is a critical complication that often occurs within 24 h after the implantation but can occur later. We report a case of cardiac perforation of the right ventricular wall during the chronic period, 2 years after pacemaker implantation.

## Abstract 2

We should recognize the possibility that a cardiac perforation and tamponade caused by the lead of cardiac implantable electronic devices can occur even years after implantation, despite it being known to occur within 24 h of implantation.

## 1 INTRODUCTION

Cardiac perforation by the lead of cardiac implantable electronic devices (CIEDs) has a reported incidence of 0.1%–5.2% after pacemaker and implantable cardioverter‐defibrillator implantation.^1^, ^2^ It is a rare but critical complication, because it results in cardiac tamponade or pacing failure.^2^ Emergency surgery is necessary when right ventricular (RV) perforation is suspected. Usually, it occurs within 24 h after the implantation, but a few cases that occurred during the chronic period have been reported. Herein, we report a case of cardiac perforation of the RV wall without infection during the chronic period, 2 years after pacemaker implantation.

## 2 CASE PRESENTATION

A 67‐year‐old woman, who underwent pacemaker implantation due to sick sinus syndrome at our hospital 2 years, previously presented to our outpatient clinic with severe chest pain and palpitations (atrial lead, BOSTON 7735–45 cm (active fixation); ventricular lead, BOSTON 7742–59 cm (active fixation); generator, BOSTON ACCOLADE MRI EL DR/L331; Boston Medical, Shrewsbury, MA, USA). There was no pacing failure after the perforation because the pacemaker setting was mostly atrial pacing and ventricle sensing (99%). Her chronic RV lead threshold was 1.4 V at 0.4 ms, and the impedance was 682 ohms. Initial examination showed no abnormality, but she was admitted for severe pain. The next day, cardiac perforation was suspected from chest X‐ray and computed tomography (CT) findings, and she was referred to our department. She had no history of collagen disease or steroid use.

**FIGURE 1:**
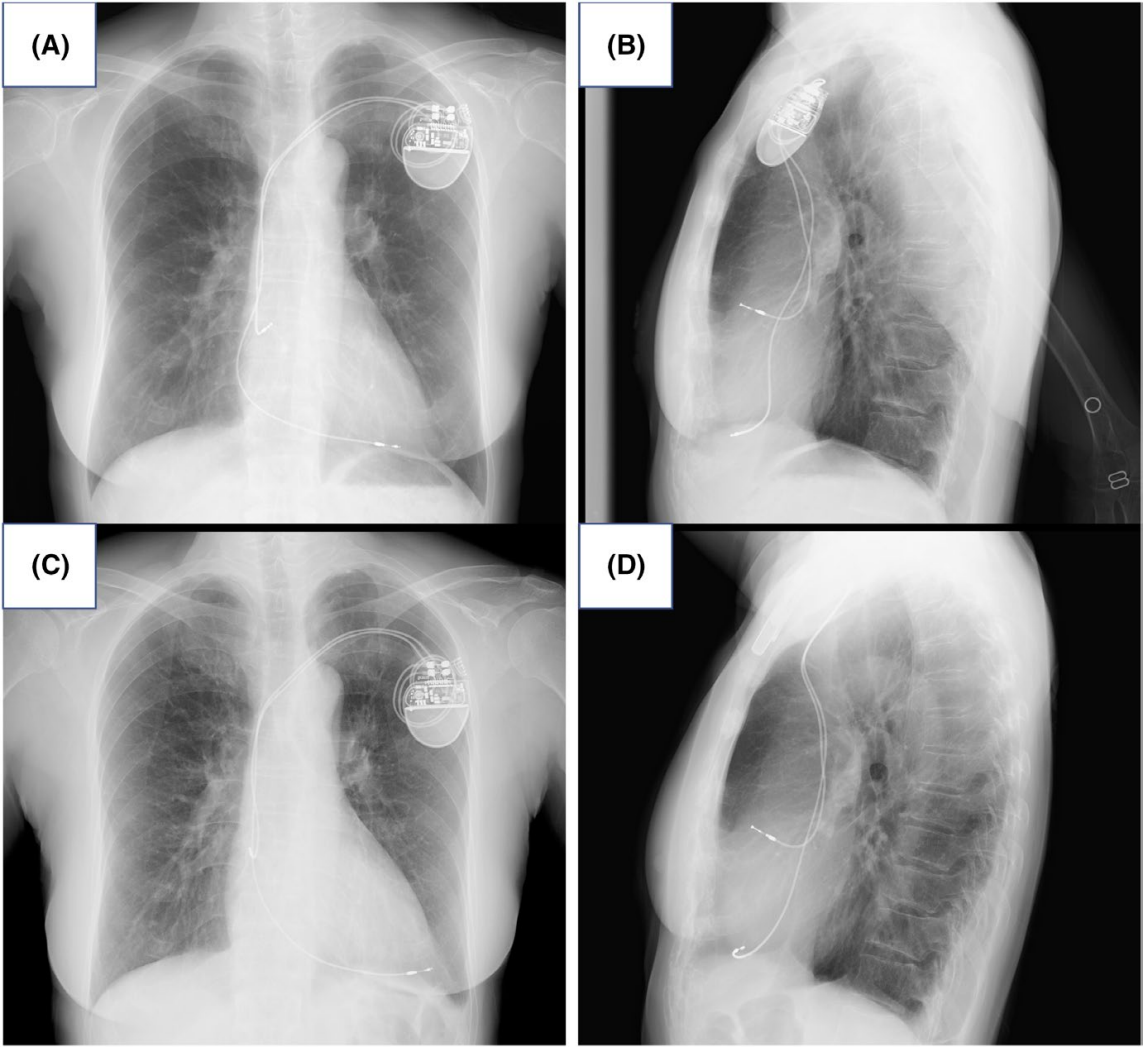
Chest X‐ray showing the tip of the RV lead had moved on the right ventricular apex more than the day before. (A) (B) Films obtained at the time of the outpatient visit presented severe chest pain. (C) (D) Films obtained at the next day of admission

**FIGURE 2:**
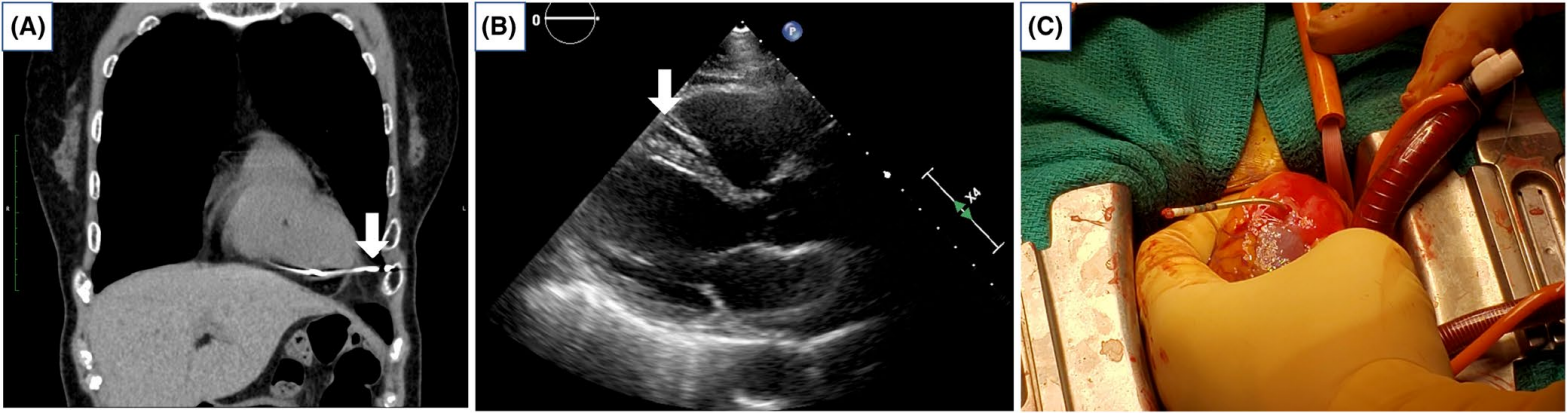
(A) Chest computed tomography image showing the apically sited right ventricular lead perforating through the right ventricle (arrow). (B) Echocardiogram revealed no findings of cardiac tamponade. The lead staying in the right ventricle(arrow). (C) RV lead had perfectly perforated the right ventricular wall about 3 cm

Her blood pressure was 148/78 mmHg, and the heart rate was 64 bpm. Laboratory tests revealed hemoglobin concentration of 14.1 g/dL, white blood cell count of 7000 cells/μL, and C‐reactive protein of 0.53 mg/dL. The RV lead threshold increased to 3.5 V at 2.0 ms, and the impedance value decreased to 541 ohms. There was no change in the QRS waveform of ventricular pacing because the ventricle pacing ratio was below 1%. Chest X‐ray imaging showed that the tip of the RV lead had moved further onto the RV apex than the day before (Figure 1). The chest CT seemed to show the apically located RV lead perforating the right ventricle, but the echocardiogram revealed no findings suggesting cardiac tamponade (Figure 2).

We believed that emergency surgery was unnecessary, because there were no findings of cardiac tamponade and her symptoms improved. We planned the operation for 3 days after admission.

The operation was performed by median sternotomy. There were no findings of adhesion or infection in the pericardium. We confirmed that the RV lead had perforated the RV wall by about 3 cm (Figure 2C), and a small amount of homolid pericardial fluid was detected in the pericardium. We repaired the RV wall and disconnected the old RV lead using cardiopulmonary bypass. It was possible to simply remove the ventricular lead, but a new ventricular lead was implanted in the posterior RV just in case. The patient was discharged 13 days after the operation. The patient continues to do well about 1 year after the surgery.

## 3 DISCUSSION

RV perforation has a reported incidence of 0.3%–1.2% post‐pacemaker implantation and 0.6%–4.2% post‐implantable cardioverter‐defibrillator implantation. The incidence of RV perforation is greater in older patients (>80 years) or those who have a low (<20 kg/m^2^) or high body mass index (>30 kg/m^2^).^1^ Cardiac perforations tend to be more prevalent in active‐fixation leads because the helical screwing mechanism is prone to penetration through the myocardium. However, a recent population‐based cohort study performed in Taiwan reported no difference in the risk of cardiac perforation between active‐ and passive‐fixation pacing leads.^3^ Various symptoms were presented, for example, chest pain, shortness of breath, and cardiac shock, with some cases being asymptomatic. Transthoracic echocardiography and CT are useful for definitive diagnosis. As many as 15% of patients with CT‐confirmed delayed perforation are asymptomatic.^4^ Transthoracic echocardiography can also be helpful for the detection of pacing wire perforation when the path of the wire is visualized in the spatial orientation of the echocardiography beam. Pericardial effusion detected using echocardiography can be a sign of lead perforation, but other mechanisms can cause effusion, such as traumatic inflammation of the myocardium and pericardium from the lead screw, or irritation of the visceral pericardium by immune‐mediated mechanisms.^5^, ^6^ In this case, no findings were suggestive of cardiac tamponade on CT, and there was no pericardial fluid detected on echocardiography.

Usually, perforation occurs within 24 h after the implantation (76%), and it rarely occurs more than 1 week post‐implantation.^7^ Perforation that occurs after more than 1 month is defined as delayed perforation. In the studies we analyzed, most RV perforations during the chronic period occurred within 1 month, and there are few reports of perforation after more than 1 year. Transvenous lead extraction may be performed in the electrophysiology laboratory with continuous electrocardiographic and arterial bleed pressure monitoring depending on the patient's general condition and the availability of a cardiac surgery team and an operating room.^8^ However, when tined leads perforate the myocardium and possibly the pericardium, there is a major concern that the bulky tip of the lead can damage tissues during removal. Fibrosis around the lead tip increases the risk of tissue damage with transvenous extraction.^9^ Severe RV damage by the CIED lead tip is more likely to be fatal, requiring emergency surgery in many cases because of the risk of death from tamponade. Therefore, we were concerned that delayed timing of perforation (>1 month) and the use of an active‐fixation lead would require surgical extraction.

In this case, we learned some important lessons. Emergency surgery should always be considered whenever RV perforation is suspected. We believed that an emergency operation was not necessary because 2 years had elapsed after pacemaker implantation and there was no sign of infection. Moreover, we considered that the incomplete perforation was caused by the continuous compression of the right ventricle by the lead. We predicted that bleeding was unlikely because of the adhesion in the perforated area and an elective operation was performed. However, in hindsight, we should have performed emergency surgery because there was no adhesion and this created a risk of tamponade. It is desirable to consider urgent thoracotomy when RV perforation is suspected, even if considerable time has elapsed since pacemaker implantation, because such perforation can cause cardiac tamponade.

## CONFLICTS OF INTERESTS

None.

## AUTHOR CONTRIBUTIONS

Akihiro Yamamoto produced the manuscript, edited the figures, provided revisions, and liaised with the publisher. Shoichi Takahashi helped produce the manuscript and provided revisions on a different version of the manuscript.

## ETHICAL APPROVAL

Approval of the International Review Board was not required at our institution because this study was a case report, and informed consent was obtained from the patient for publication.

## CONSENT

Written informed consent was obtained from the patient to publish this report in accordance with the journal's patient consent policy.

## PERMISSION TO REPRODUCE MATERIAL FROM OTHER SOURCES

None.

## CLINICAL TRIAL REGISTRATION

None.

## Data Availability

The data that support the findings of this study are available from the corresponding author upon reasonable request.

## References

[1] Udo EO , Zuithoff NPA , van Hemel NM , et al. Incidence and predictors of short‐ and long‐term complications in pacemaker therapy: the FOLLOWPACE study. Heart Rhythm. 2012;9:728‐735. 22182495 10.1016/j.hrthm.2011.12.014

[2] Rav Acha M , Rafael A , Keaney JJ , et al. The management of cardiac implantable electronic device lead perforations: a multicentre study. Europace. 2019;21:937‐943. 31157389 10.1093/europace/euz120

[3] Lin YS , Chen TH , Hung SP , et al. Impact of pacemaker lead characteristics on pacemaker related infection and heart perforation: a nationwide population‐based cohort study. PLoS One. 2015;10:e0128320. 26075602 10.1371/journal.pone.0128320 PMC4468132

[4] Hirschl DA , Jain VR , Spindola‐Franco H , Gross JN , Haramati LB . Prevalence and characterization of asymptomatic pacemaker and ICD lead perforation on CT. Pacing Clin Electrophysiol. 2007;30:28‐32. 17241311 10.1111/j.1540-8159.2007.00575.x

[5] Akbarzadeh MA , Mollazadeh R , Sefidbakht S , Shahrzad S , Bafruee NB . Identification and management of right ventricular perforation using pacemaker and cardioverter‐defibrillator leads: a case series and mini review. J Arrhythm. 2017;33:1‐5. 28217220 10.1016/j.joa.2016.05.005 PMC5300868

[6] Khalid M , Murtaza G , Ayub MT , Ramu V , Paul T . Right ventricle perforation post pacemaker insertion complicated with cardiac tamponade. Cureus. 2018;10:e2266. 29736351 10.7759/cureus.2266 PMC5935430

[7] Cano Ó , Andrés A , Alonso P , et al. Incidence and predictors of clinically relevant cardiac perforation associated with systematic implantation of active‐fixation pacing and defibrillation leads: a single‐centre experience with over 3800 implanted leads. Europace. 2017;19:96‐102. 26847075 10.1093/europace/euv410

[8] Zhou X , Ze F , Li D , Wang L , Guo J , Li X . Outcomes of transvenous lead extraction in patients with lead perforation: a single‐center experience. Clin Cardiol. 2020;43:386‐393. 31904110 10.1002/clc.23327 PMC7144486

[9] Demo H , Megaly MM . Late perforation of a passively fixated pacemaker lead through the right ventricle. a report and review of literature. J Cardiol Cases. 2017;16:148‐150. 30279821 10.1016/j.jccase.2017.07.002 PMC6149285

